# A peptide derived from TID1S rescues frataxin deficiency and mitochondrial defects in FRDA cellular models

**DOI:** 10.3389/fphar.2024.1352311

**Published:** 2024-03-01

**Authors:** Yi Na Dong, Lucie Vanessa Ngaba, Jacob An, Miniat W. Adeshina, Nathan Warren, Johnathan Wong, David R. Lynch

**Affiliations:** ^1^ Department of Pediatrics and Neurology, The Children’s Hospital of Philadelphia, Philadelphia, PA, United States; ^2^ Department of Neurology, Perelman School of Medicine, University of Pennsylvania, Philadelphia, PA, United States

**Keywords:** Friedreich ataxia (FRDA), frataxin, Tid1, mitochondrial fragmentation, ATP

## Abstract

Friedreich’s ataxia (FRDA), the most common recessive inherited ataxia, results from homozygous guanine–adenine–adenine (GAA) repeat expansions in intron 1 of the *FXN* gene, which leads to the deficiency of frataxin, a mitochondrial protein essential for iron-sulphur cluster synthesis. The study of frataxin protein regulation might yield new approaches for FRDA treatment. Here, we report tumorous imaginal disc 1 (TID1), a mitochondrial J-protein cochaperone, as a binding partner of frataxin that negatively controls frataxin protein levels. TID1 interacts with frataxin both *in vivo* in mouse cortex and *in vitro* in cortical neurons. Acute and subacute depletion of frataxin using RNA interference markedly increases TID1 protein levels in multiple cell types. In addition, TID1 overexpression significantly increases frataxin precursor but decreases intermediate and mature frataxin levels in HEK293 cells. In primary cultured human skin fibroblasts, overexpression of TID1S results in decreased levels of mature frataxin and increased fragmentation of mitochondria. This effect is mediated by the last 6 amino acids of TID1S as a peptide made from this sequence rescues frataxin deficiency and mitochondrial defects in FRDA patient-derived cells. Our findings show that TID1 negatively modulates frataxin levels, and thereby suggests a novel therapeutic target for treating FRDA.

## 1 Introduction

Friedreich’s ataxia (FRDA) is an autosomal recessive neurodegenerative disease characterized by progressive gait and limb ataxia, hypertrophic cardiomyopathy, scoliosis, slurred speech, vision loss and in some individuals diabetes (Strawser et al., 2017). FRDA is typically caused by homozygous, expanded guanine–adenine–adenine (GAA) repeats in the intron 1 of frataxin gene leading to transcriptional silencing of frataxin gene (Campuzano et al., 1996). The mitochondrial protein frataxin is essential for the formation of iron-sulfur clusters (ISC). Frataxin deficiency reduces the activity of ISC-containing enzymes such as aconitase and respiratory complex II (Rötig et al., 1997; Stehling et al., 2004), resulting in impaired ATP synthesis and mitochondrial respiration (Lodi et al., 1999; Zarse et al., 2007) as well as increased mitochondrial iron and oxidative stress (Puccio et al., 2001; Al-Mahdawi et al., 2006), and cause pathological changes in affected tissues. Increasing frataxin levels is therefore the most direct approach for FRDA treatment.

Tumorous imaginal disc 1 (TID1), also called DnaJ homolog subfamily A member 3, mitochondrial (DNAJA3), is a member of the heat shock protein (Hsp) 40 family. TID1 interacts with the Hsp70 family of chaperone proteins through its distinctive J domain, a highly conserved tetrahelical region, which increases their ATPase activity for substrate binding and functions as a cochaperone and regulatory component for Hsp70 (Hendrick et al., 1993; Silver and Way, 1993; Syken et al., 1999; Trentin et al., 2001; Liu et al., 2012). TID1 also affects cell survival, proliferation, and responses to stress (Cheng et al., 2001; Syken et al., 2003; Tarunina et al., 2004; Lo et al., 2004; Chen et al., 2009). TID1 null mutations are lethal (Lo et al., 2004). In mice, a particular TID1-related cardiac deficiency also leads to progressive respiratory chain deficiency, dilated cardiomyopathy, and premature death before the age of 10 weeks (Hayashi et al., 2006). TID1 encodes two mitochondrial matrix localized splice variants, TID1-long (TID1L, 43 kDa) and -short (TID1S, 40 kDa), which differ only at their carboxyl termini (Lu et al., 2006). The cellular targets and functions of TID1L and TID1S are distinct. For instance, TID1S interacts with the agrin receptor and is involved in neuromuscular transmission, whereas TID1L connects with the von Hippel-Lindau protein and is implicated in tumor suppression (Linnoila et al., 2008; Bae et al., 2005). TID1L enhances external stimulus-induced apoptosis, whereas TID1S suppresses it (Syken et al., 1999).

The mitochondrial molecular chaperone GRP75 regulates frataxin post-translationally (Dong et al., 2019). It is unknown if the cochaperone TID1 regulates frataxin. In this study, we examined the physical and functional interactions between frataxin and TID1 using cellular and molecular approaches and suggest that TID1S negatively regulates frataxin levels. A peptide derived from the last six amino acids of TID1S rescues frataxin deficiency and mitochondrial defects in FRDA patient-derived cells. These findings demonstrate that TID1S is a novel regulator of frataxin and thus provide a new therapeutic target for FRDA.

## 2 Materials and methods

### 2.1 Animals

C57BL/6 mice (stock no: 000664) were purchased from Jackson Laboratory. Doxycycline inducible frataxin knockdown (FRDAkd) mice were originally obtained from Drs. Geschwind and Chandran at University of California (Los Angeles, CA), then bred with C57BL/6 mice (The Jackson Laboratory Stock No: 000664) to generate wild type (WT) and transgenic (TG) mice. All mice were housed in an environment of 12 h light/dark cycle, temperature of 25 ± 2°C, 55% humidity, with *ad libitum* standard diet and water; treated according to the protocols approved by the Children’s Hospital of Philadelphia Institutional Animal Care and Use Committee (IACUC; protocol 16-250); and genotyped at weaning by commercial vendor (Transnetyx, Cordova, TN). Wildtype and transgenic mice were fed Dox-compounded chow diet (200PPM Doxycycline, Animal Specialties and Provisions, LLC., Quakertown, PA) to induce frataxin knockdown in the transgenic mice. Mice were harvested at 4 weeks after doxycycline treatment.

### 2.2 Preparation of tissue homogenates

Mice were deeply anesthetized and euthanized by decapitation. The cerebellum, heart and skeletal muscle were collected and homogenized with a Potter-Elvehjem homogenizer (Thermo Fisher Scientific Inc., Hampton, NH) in RIPA buffer (100 mM Tris-HCL, 150 mM NaCl, 1% IGEPAL, 1 mM EDTA, pH 7.4) containing a protease inhibitor cocktail (1:500 dilution; Calbiochem, Darmstadt, Germany). The homogenates were spun down at 13,000 rpm for 15 minutes (min). The supernatant was frozen and kept at −80°C until it was used for Western blot or Co-Immunoprecipitation.

### 2.3 Preparation of primary neuronal cultures

Primary rat cortical neurons were derived from embryonic day 17 Sprague Dawley rat embryos, as described previously (Dong et al., 2019). Cells were used after at least 14 days *in vitro*.

### 2.4 RNA interference-mediated downregulation of frataxin in human skin fibroblasts and overexpression of plasmid DNAs in HEK293 cells

Human skin fibroblasts (Dr. Marek Napierala, UT Southwestern Medical Center, Dallas, Texas) were cultured as described previously (Dong et al., 2019). Frataxin knockdown was achieved by transfection of human frataxin siRNA (5′-rGrArArCrCrUrArUrGrUrGrArUrCrArArCrArArGrCrArGAC-3′) (Integrated DNA Technologies, Coralville, IA) into human skin fibroblasts using Lipofectamine RNAiMax reagent (Thermo Fisher Scientific, Hampton, NH). TID1L siRNA sequence is as follows: 5′-GAA​GCA​AGG​CTA​GGC​GTG​A-3′ (Ng et al., 2014). Scrambled siRNA was used as a negative control. Cells were collected after 3 days.

For overexpression of plasmid DNAs, HEK293 cells were cultured and transfected as described previously (Dong et al., 2019). Briefly, plasmid DNAs containing wild-type frataxin fused to a C-terminal HA tag, frataxin G130V mutant fused to a C-terminal HA tag (Clark et al., 2017), wild-type TID1L, wild-type TID1S, TID1L H121Q, TID1S H121Q (Both wildtype and mutants are from Addgene, Cambridge, MA), TID1S-Flag (Genscript, Piscataway, NJ) were transfected into HEK 293 cells using LipofectamineTM 2000 reagent (Thermo Fisher Scientific, Hampton, NH) for 24 h. Cells were then collected and subjected to Western blotting or immunofluorescence.

### 2.5 Lentiviral transduction of human skin fibroblasts

Human skin fibroblasts from healthy individuals and FRDA patients were cultured as described previously (Dong et al., 2022). Lentivirus containing the pHAGE-TID1L gene (Genscript, Piscataway, NJ), pHAGE-TID1S gene (genscript, Piscataway, NJ), or vector control (pHAGE-CMV-dsRed-UBC-GFP-W) (Addgene, Cambridge, MA 02139), made at the research vector core at the Children’s Hospital of Philadelphia, was transduced into cultured fibroblasts in the presence of polybrene (8 μg/mL) (Sigma, St. Louis, MO). The medium was changed to normal culture medium after 1 day transduction. Fibroblasts were collected after 5 days transduction and used for experiments.

### 2.6 Co-immunoprecipitation

Cultured rat cortical neurons or HEK293 cells were rinsed with PBS and lysed in RIPA buffer (100 mM Tris-HCL, 150 mM NaCl, 1% IGEPAL, 1 mM EDTA, pH 7.4) with a protease inhibitor cocktail (1:500 dilution; Calbiochem, Darmstadt, Germany) at 4°C for 1 h. Cell lysates were then spun down at 13,000 rpm for 10 min and the supernatant was collected and used in the assay. Co-immunoprecipitation was performed as described previously (Dong et al., 2019). The following antibodies were used: TID1L (Abcam, Cambridge, MA), TID1L/S (Santa Cruz Biotechnology, Dallas, TX), or MPP (Proteintech, Rosemont, IL).

### 2.7 Platelets and Peripheral blood mononuclear cells isolation

Whole blood collected from both healthy individuals and FRDA patients were spun at 800 rpm for 10 min. Platelet-rich plasma were then transferred to microcentrifuge tube and spun at 10,000 g for 3 min. Platelets were washed in PBS twice followed by sonication (Output: 3; Duty Cycle: 50%; 20 pulses) in RIPA buffer (See above) and centrifugation (13,200 rpm × 3 min). The supernatant was stored at −80° until use.

For peripheral blood mononuclear cells (PBMCs) isolation, whole blood was diluted with PBS at 1:1 ratio before added to the top of 8 mL RT Ficoll-Paque (GE Healthcare, Chicago, IL). Blood samples were then spun at 1,600 rpm for 25 min (room temperature, no brake) in swing bucket centrifuge. PBMCs layer was transferred to a new tube and diluted with PBS (30–50 mL volume). After 15 min centrifugation at 1,000 rpm (room temperature, high brake), the supernatant was aspirated out and PBMCs were resuspened and washed in PBS twice before lysed in RIPA buffer (see above) for future use.

### 2.8 Western blot

Cultured human fibroblasts and HEK293 cells were collected in Laemmli sample buffer (50 mM Tris-HCl, pH 6.8, 2% SDS, 5 mM EDTA, 0.1% bromophenol blue, 10% glycerol, and 2% β-mercaptoethanol). Buccal cells from controls and FRDA patients were collected in extraction buffer (Abcam, Cambridge, MA) as previously described (Deutsch et al., 2010) and diluted with Laemmli sample buffer. A sample (15–50 μg) of total protein was boiled for 5 min and then loaded onto NuPAGE 4%–12% Bis-Tris gel. Following SDS-PAGE, proteins were transferred to nitrocellulose, blocked with 3% dry milk, and incubated with antibodies against: frataxin (Abcam, Waltham, Boston), TID1L/S (Santa Cruz Biotechnology, Dallas, TX), TID1L (Abcam, Waltham, Boston), actin (Sigma, St. Louis, MO), HA (Cell Signaling, Danvers, MA), actin (Cell signaling, Danvers, MA), Aconitase 2 (Abcam, Waltham, Boston), GRP75 (Abcam, Waltham, Boston), TOM20 (Abcam, Waltham, Boston). Blots were then incubated with appropriate horseradish peroxidase-conjugated secondary antibodies and developed using enhanced chemiluminescence (Pierce, Rockford, IL).

### 2.9 Mitochondria isolation

Mitochondria were isolated from HEK293 cells transfected with frataxin-HA, TID1L or TID1S plasmid DNAs using mitochondria isolation kit (Thermo Fisher Scientific, Hampton, NH) and then lysed in RIPA buffer (See above) before Western blot analysis.

### 2.10 Immunofluorescence

Immunofluorescence was performed as previously described (Dong et al., 2019). Mitotracker™ Red CMXRos (Thermo Fisher Scientific, Waltham, MA) was loaded to human skin fibroblasts for 45 min before immunofluorescence was performed. The following antibodies were used: HA (Cell Signaling, Danvers, MA), Flag (Sigma, St. Louis, MO), TID1L (Abcam, Waltham, Boston), TID1L/S (Santa Cruz Biotechnology, Dallas, TX), GFP (Neuromab, Davis, CA at 1/200).

### 2.11 Peptide treatment

Human skin fibroblasts were treated with TID1S448-453Tat (KRSTGNYGRKKRRQRRR) (Genscript, Piscataway, NJ) or scrambled TID1S448-453Tat (SNRTKGYGRKKRRQRRR) (Genscript, Piscataway, NJ) for 48 h followed by Western blot analysis or Immunoflurescence.

### 2.12 Determination of ATP content

An equal number of skin fibroblasts transduced with lentivirus containing pHAGE-TID1L or vector control were seeded in a 96-well plate. ATP content of the cells was quantified with the CellTiter-Glo Luminescent Cell Viability Kit (catalog # G7570, Promega, Madison, WI).

### 2.13 *In vitro* binding assay

2 μg Glutathione-S-Transferase (GST) (Sigma, St. Louis, MO) or recombinant human frataxin fused to an N-terminal GST tag (GST-Frataxin) (Novus biologicals Littleton, CO) were bound to glutathione beads in RIPA buffer (100 mM Tris-HCL, 150 mM NaCl, 1% IGEPAL, 0.5% Sodium deoxycholate, 1 mM EDTA, pH 7.4) overnight at 4°C. After 4 washes, 2 μg human TID1L fused to a C-terminal Myc/DDK tag (Origene, Rockville, MD, Catalog #TP315039) was added and incubated overnight at 4°C. Glutathione beads were then washed 3 times, eluted with Laemmli sample buffer, and subjected to SDS-PAGE.

### 2.14 Statistical analysis

Calculations of statistical differences were assessed by Two-tailed Student’s t-test and a probability value of *p* <0.05 was considered statistically significant.

## 3 Results

### 3.1 TID1 physically interacts with frataxin both in mouse brain cortex and neuronal cells

**FIGURE 1:**
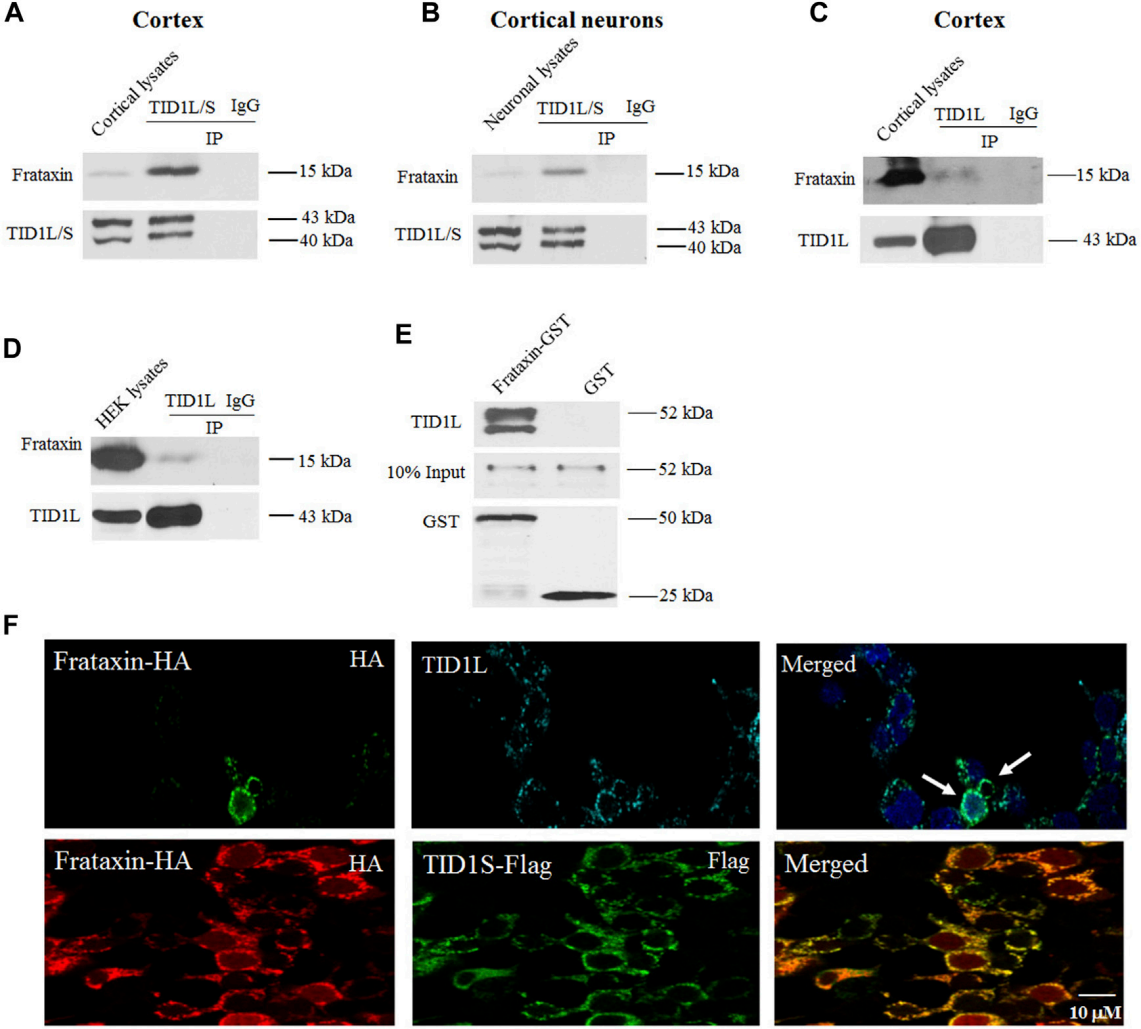
TID1 physically interacts with frataxin both *in vivo* in mouse cortex and *in vitro* in cortical neurons. In both cortical homogenates **(A)** and neuronal lysates **(B)**, frataxin was immunoprecipitated by a TID1L/S antibody but not control IgG. Similarly, a specific TID1L antibody but not control IgG immunoprecipitated frataxin from cortical homogenates **(C)** and HEK293 cells **(D)** but to a much lesser extent. In an *in vitro* binding assay, purified glutathione S- transferase (GST)-frataxin but not purified GST pulled down TID1L **(E)**. In HEK293 cells transfected with frataxin-HA and TID1S-Flag, immunofluorescence was performed using anti-HA and anti-Flag antibodies. Frataxin colocalized with TID1S **(F)**. Frataxin also colocalized with endogenously expressed TID1L stained with an anti-TID1L antibody **(F)**, further supported the interaction between TID1 and frataxin.

Frataxin was immunoprecipitated using an anti-frataxin antibody in mouse cortical homogenates, followed by mass spectrometry analysis in order to look for binding partners to frataxin in the brain (Dong et al., 2022). TID1 was identified as a potential frataxin binding partner with three peptide fragments precipitated (Supplementary Table S1). Co-immunoprecipitation was carried out in both mouse cortical homogenates and cultured mouse cortical neurons to verify the interaction between frataxin and TID1. An antibody that recognizes both the long and short forms of TID1 (TID1L/S) precipitated frataxin in both preparations while control IgG had no effect (Figures 1A, B). These results indicate that TID1 physically interacts with frataxin both *in vivo* in mouse cortex and *in vitro* in mouse cortical neurons.

To distinguish the binding of frataxin to TID1L and TID1S, an antibody to the TID1L C-terminus (amino acids 450–480) was utilized in the Co-IP assay. TID1L antibody also pulled down frataxin in mouse cortical homogenates, but to a much lesser extent than TIDL/S antibody (Figure 1C). The same outcome was seen in HEK293 cells (Figure 1D), suggesting that TID1S may be the main form interacting with frataxin. Immunofluorescence performed in HEK293 cells transfected with frataxin-HA and TID1S-Flag plasmids also revealed the colocalization of TID1S and frataxin (Figure 1F). Similar results were found for frataxin and endogenously expressed TID1L, further supporting the presence of physical interactions between TID1 and frataxin.

To determine whether TID1 directly interacts with frataxin, an *in vitro* binding assay was performed using purified glutathione S-transferase (GST)-frataxin fusion proteins and TID1L fused to a C-terminal C-Myc tag. GST-frataxin but not GST bound to TID1L in the *in vitro* binding assay (Figure 1E), indicating that TID1L directly interacts with frataxin.

### 3.2 Frataxin deficiency causes opposing changes in TID1 proteins

**FIGURE 2:**
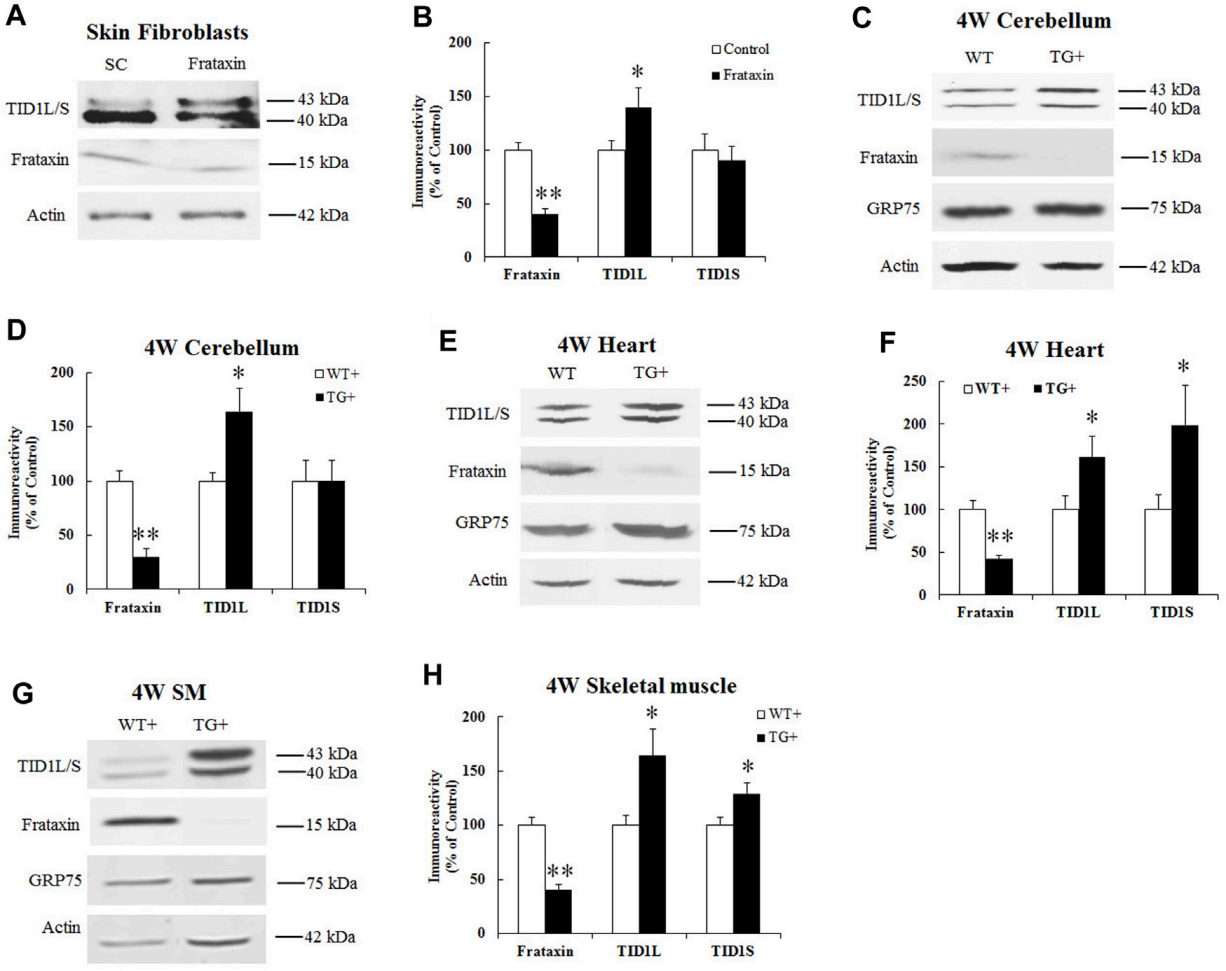
Effect of frataxin knockdown on TID1 protein levels. Representative blots and bar graph show decreased frataxin and increased TID1 protein levels in human skin fibroblasts transfected with frataxin siRNA for 3 days **(A,B)** (*n* = 6) as well as in the homogenates of cerebellum **(C,D)** (*n* = 6), heart **(E,F)** (*n* = 6) and skeletal muscle **(G,H)** (*n* = 6) from frataxin knockdown mice induced with doxycycline for 4 weeks. *p < 0.05, **p < 0.01. Data were shown as mean ± SE.

To examine whether frataxin deficiency affects TID1 protein levels, human skin fibroblasts were transfected with frataxin siRNA followed by Western blot analysis. Frataxin siRNA treatment for 3 days led to 40% residual frataxin compared with control siRNA (Figures 2A, B) (*n* = 6, *p* < 0.05), accompanied by a significant increase in TID1L protein levels (Figures 2A, B) (40% increase, *n* = 6, *p* < 0.05). Similar result was found for TID1L using TID1L antibody (Supplementary Figures S1A, B). No change was detected for TID1S. We next investigated TID1 protein levels *in vivo* in doxycycline-inducible frataxin knockdown mice. In comparison with wildtype (WT) mice, doxycycline induction for 4 weeks led to 30%, 42% and 40% residual frataxin in the homogenates of cerebellum (Figures 2C, D, *n* = 6, *p* < 0.01), heart (Figures 2E, F, *n* = 6, *p* < 0.01) and skeletal muscle (Figures 2G, H, *n* = 6, *p* < 0.01) of frataxin knockdown mice, respectively. Accordingly, TID1 proteins were also significantly increased in the homogenates of cerebellum (Figures 2C, D, 64% increase for TID1L, *n* = 6, *p* < 0.05), heart (Figures 2E, F, 62% and 99% increase for TID1L and TID1S, respectively, *n* = 6, *p* < 0.05) and skeletal muscle (Figures 2G, H, 64% and 29% increase for TID1L and TID1S, respectively, *n* = 6, *p* < 0.05) of frataxin knockdown mice. Similar results were found for TID1L in cerebellum (Supplementary Figures S1C, D) and heart (Supplementary Figures S1E, F) using TID1L antibody. GRP75, a major mitochondrial chaperone, showed no change in the homogenates of either tissue (Figures 2C, E, G). Acute frataxin knockdown also has no effect on GRP75 protein levels in human skin fibroblasts (Dong et al., 2019). These results indicate that acute and subacute frataxin deficiency specifically increases TID1 protein levels.

**FIGURE 3:**
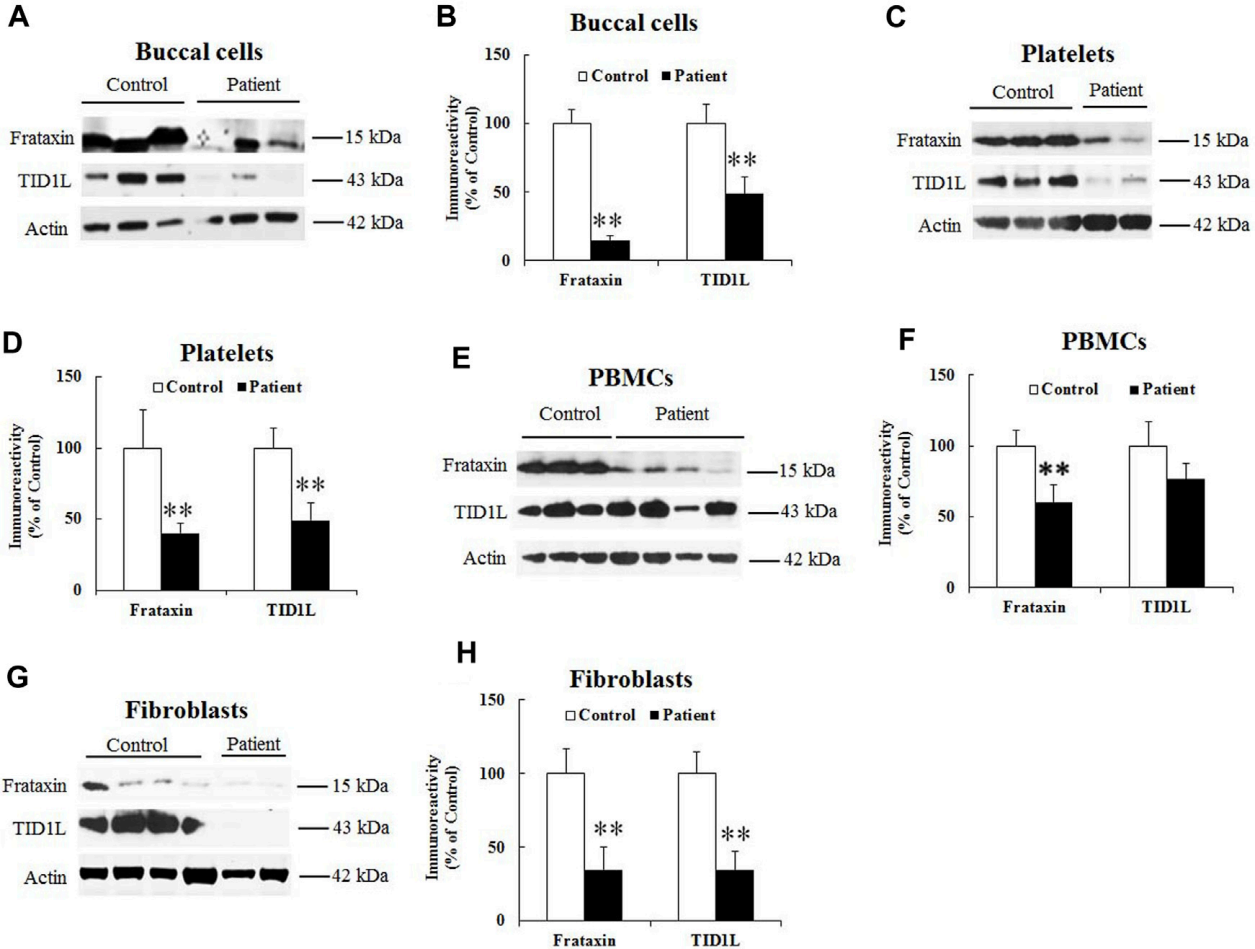
TID1L protein levels are reduced in FRDA patient-derived cells. FRDA patient buccal cells, skin fibroblasts, platelets or PBMCs were lysed and subjected to Western blotting with the indicated antibodies. The amount of immunoreactivity in the lysates was quantified as a percentage of the controls. Representative blots and bar graphs demonstrate reduced frataxin and TID1L in buccal cells **(A,B)** (*n* = 14 for controls and *n* = 16 for patients), platelets **(C,D)** (*n* = 16 for controls and *n* = 17 for patients) and fibroblasts **(G,H)** (*n* = 15 for controls and *n* = 20 for patients). While frataxin was significantly reduced in PBMCs, no change in TID1L was detected in PBMCs **(E,F)** (*n* = 18 for both controls and patients). ∗*p* < 0.05; ∗∗*p* < 0.01. Data were shown as mean ± SE.

We then examined TID1 protein in FRDA patient-derived cells. Due to the low expression level of TID1S in buccal cells, platelets and PBMCs (Supplementary Figure S2), only TID1L was measured in patient cells. Compared with healthy individuals, frataxin levels significantly decreased in buccal cells (Figures 3A, B, 78% decrease, *n* = 14 for control and *n* = 16 for patient, *p* < 0.01), platelets (Figures 3C, D, 60% decrease, *n* = 15 for control and *n* = 20 for patient, *p* < 0.01), PBMCs (Figures 3E, F, 47% decrease, *n* = 18 for both control and patient, *p* < 0.01) and fibroblasts (Figures 3G, H, 65% decrease, *n* = 16–17 both control and patient, *p* < 0.01). Accordingly, TID1L levels were significantly decreased in buccal cells (Figures 3A, B, 60% decrease, *n* = 14 for control and *n* = 16 for patient, *p* < 0.01), platelets (Figures 3C, D, 51% decrease, *n* = 15 for control and *n* = 20 for patient, *p* < 0.01) and fibroblasts (Figures 3G, H, 65% decrease, *n* = 16–17 both control and patient, *p* < 0.01). No change in TID1L was detected in PBMCs (Figures 3E, F). These results indicate that TID1L is reduced in FRDA patient-derived cells.

### 3.3 TID1 overexpression increases frataxin precursor but decreases intermediate and mature frataxin

**FIGURE 4:**
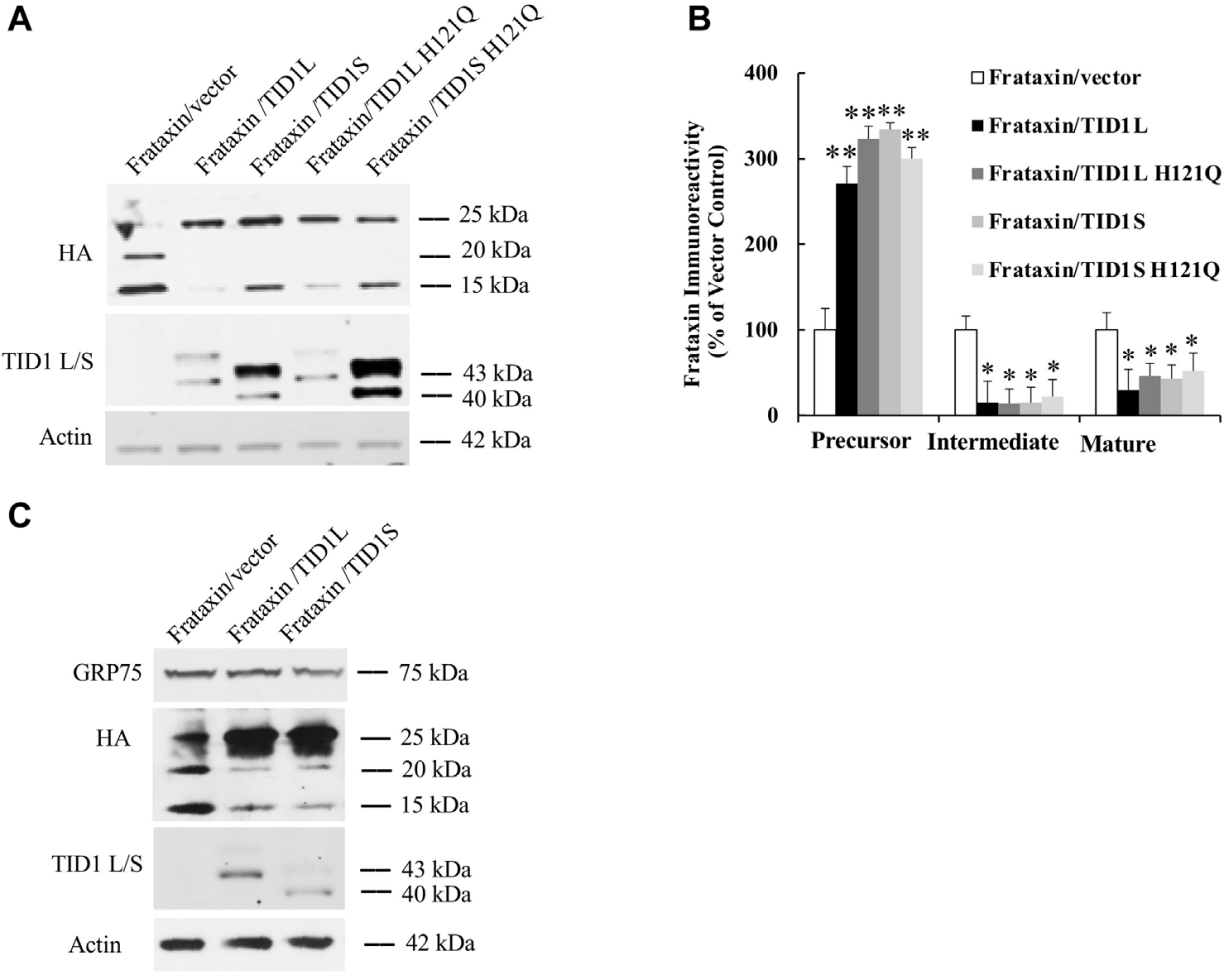
Effect of TID1 overexpression on frataxin protein levels. HEK293 cells transfected with frataxin and TID1 plasmid DNAs were lysed and subjected to Western blotting with the indicated antibodies. The amount of immunoreactivity in the lysates was quantified as a percentage of vector control. Representative blots and bar graphs demonstrate increased frataxin precursor and decreased intermediate and mature frataxin following TID1L or TID1S overexpression **(A,B)** (*n* = 8). TID1L H121Q and TID1S H121Q had similar effects as wildtype TID1L and TID1S **(A,B)** (*n* = 6), respectively. No change was found in GRP75 levels upon TID1L or TID1S overexpression **(C)**. ∗*p* < 0.05, ∗∗*p* < 0.01. Data were shown as mean ± SE.

To investigate the functional significance of TID1 elevation upon acute and subacute frataxin deficiency, TID1 protein was overexpressed in HEK293 cells along with frataxin followed by Western blot analysis. Both TID1 precursor and mature form were detected when overexpressed in HEK293 cells. Compared with vector control, both TID1L and TID1S overexpression significantly increased frataxin precursor (Figures 4A, B, 171% increase and 234% increase for TID1L and TID1S, respectively, *n* = 8, *p* < 0.01) but decreased intermediate and mature frataxin (Figures 4A, B, Intermediate frataxin: 85% decrease for both TID1L and TID1S, *n* = 8, *p* < 0.05; Mature frataxin: 70% and 57% decrease for TID1L and S, respectively, *n* = 8, *p* < 0.05). Given that GRP75 regulates frataxin (Dong et al., 2019) and TID1 is a cochaperone of GRP75 (Trentin et al., 2001), we overexpressed TID1L H121Q and TID1S H121Q, mutants functionally inactivate TID1 by allowing to bind to, but not to activate, Hsp70 chaperone proteins (Skyen et al., 1999), to rule out the potential that GRP75 mediates the impact of TID1 on frataxin. Both TID1L H121Q and TID1S H121Q overexpression had similar effects on frataxin as wildtype TID1L and TID1S (Figures 4A, B, frataxin precursor: 224% and 200% increase for TID1L H121Q and TID1S H121Q, respectively, *n* = 6, *p* < 0.01; intermediate frataxin: 85% and 78% decrease for TID1L H121Q and TID1S H121Q, respectively, *n* = 6, *p* < 0.05; mature frataxin: 57% and 48% decrease for TID1L H121Q and TID1S H121Q, respectively, *n* = 6, *p* < 0.05). TID1L and TID1S overexpression also had no effect on GRP75 levels (Figure 4C). These results indicate that TID1 regulates frataxin independently of its ability to activate GRP75.

**FIGURE 5:**
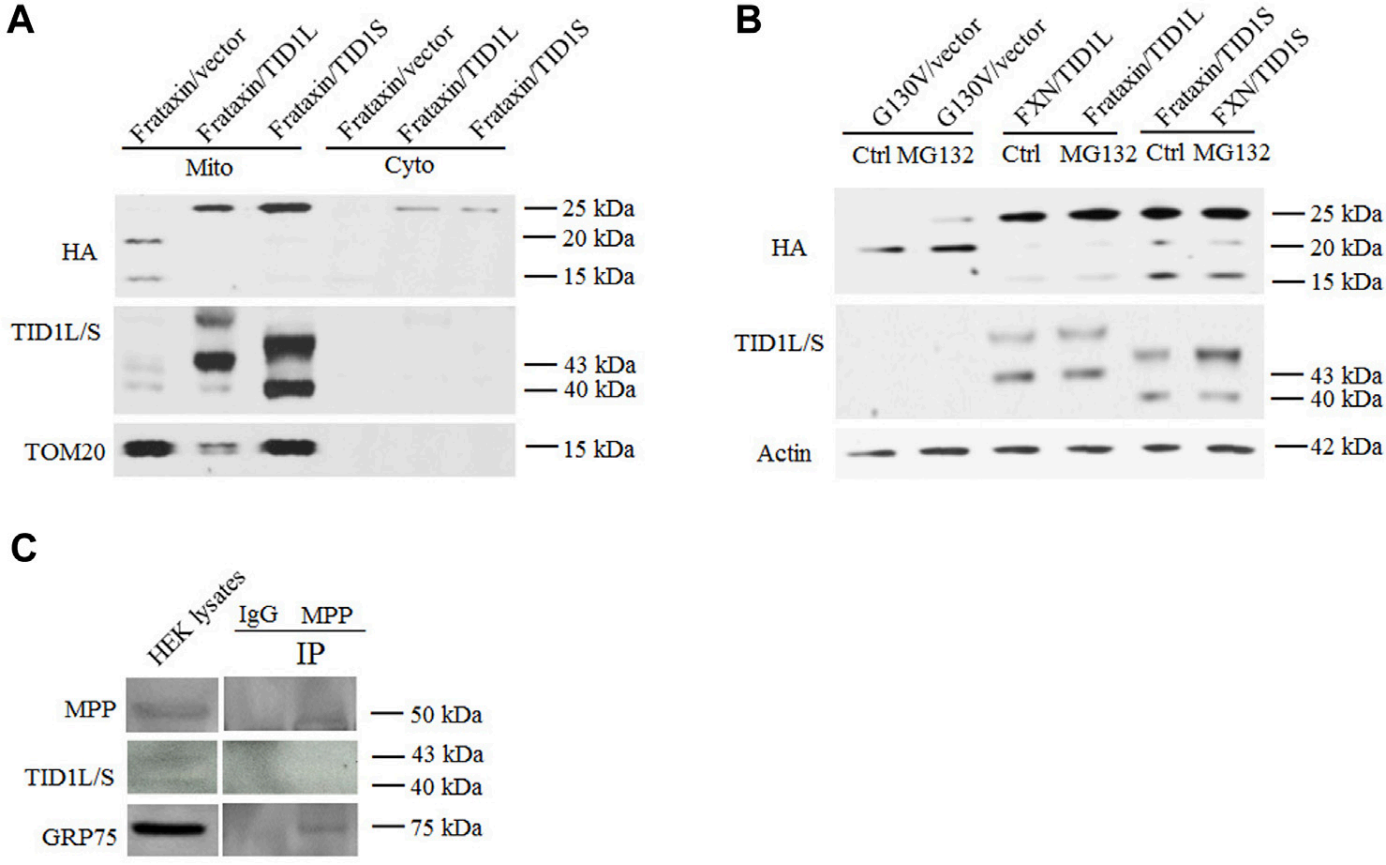
Mechanistic study of TID1 overexpression-caused changes in frataxin levels. HEK293 cells transfected with frataxin and TID1 plasmid DNAs were subject to mitochondria fractionation followed by Western blotting analysis. Frataxin precursor is predominantly localized in the mitochondrial fraction upon TID1L or TID1S overexpression **(A)**. Tom20 was used as a mitochondrial marker **(A)**. Treatment with MG132 (10 μM) also had no effect on TID1L or TID1S overexpression-caused decrease in intermediate and mature frataxin **(B)**. Frataxin G130V mutant transfected HEK293 cells were used as a positive control **(B)**. Neither TID1L nor TID1S interacted with MPP, in contrast to GRP75 **(C)**.

TID1 overexpression increases levels of frataxin precursor but decreases levels of intermediate and mature frataxin, suggesting that frataxin precursor is either not imported or is degraded. To test these hypotheses, subcellular fractionation was performed in HEK293 cells followed by Western blot analysis. As shown in Figure 5A, increased frataxin precursor caused by TID1L or TID1S overexpression were predominantly localized in the mitochondrial fraction whereas a small portion was localized in the cytosolic fraction, suggesting that decreased intermediate and mature frataxin are not caused by impaired mitochondrial import. Treatment with a proteasomal inhibitor MG132 had no effect on the levels of intermediate and mature frataxin while same treatment significantly increased frataxin G130V precursor and intermediate forms (Figure 5B), consistent with our previous report on such variant (Clark et al., 2017). These results indicate that TID1L or TID1S overexpression caused a decrease in intermediate and mature frataxin that is not caused by proteasomal degradation either.

As GRP75 regulates frataxin partly through its interaction with mitochondrial processing peptidase (MPP) (Dong et al., 2019), we next examined whether TID1L and TID1S interact with MPP using a Co-IP assay. An antibody against MPP pulled down GRP75 but not TID1L or TID1S (Figure 5C), suggesting that neither TID1L nor TID1S interact with MPP and alter frataxin processing by MPP.

### 3.4 TID1S overexpression decreases mature frataxin and ATP levels in human skin-derived fibroblasts

**FIGURE 6:**
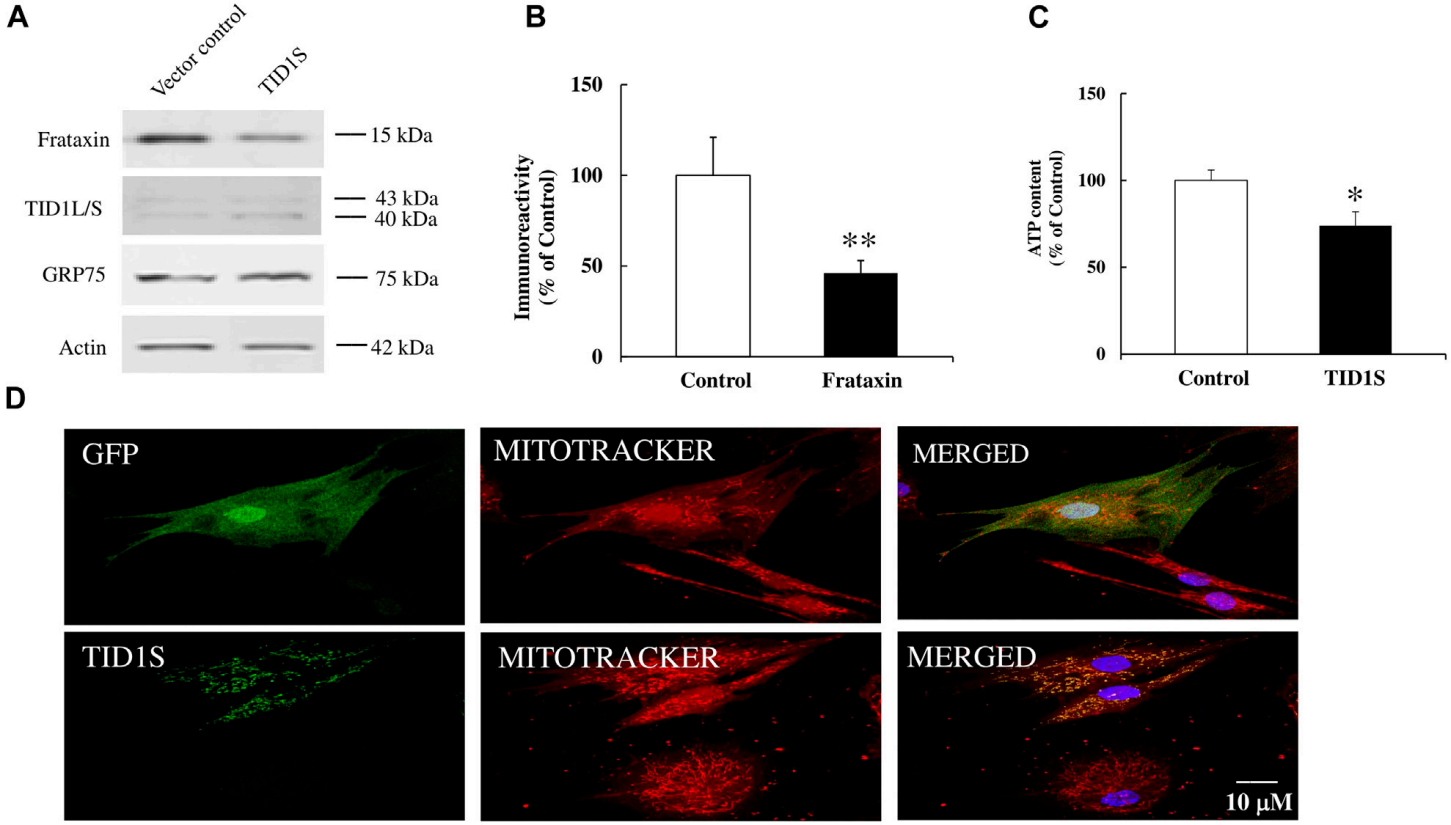
TID1S overexpression decreases mature frataxin in human skin fibroblasts. Human skin fibroblasts from healthy individuals were transduced with lentivirus carrying pHAGE-TID1S gene or vector control for 5 days before Western blotting or immunofluorescence. TID1S transduction decreased mature frataxin **(A, B)** and ATP levels **(C)** (*n* = 5). TID1S transduction also caused mitochondria fractionation in fibroblasts **(D)**. Vector control was stained with an anti-GFP antibody and TID1S was stained with an anti-TID1L/S antibody. ∗*p* < 0.05, ∗∗*p* < 0.01. Data were shown as mean ± SE.

To examine whether TID1 overexpression affects frataxin levels in primary cultured cells, lentivirus carrying pHAGE-TID1S gene or vector control was transduced into human skin fibroblasts for 5 days followed by Western blot analysis. TID1S overexpression decreased mature frataxin levels in fibroblasts (Figures 6A, B, 54% decrease, *n* = 5, *p* < 0.05) compared with vector control. Similar as in HEK293 cells, TID1S overexpression had no effect on GRP75 in fibroblasts. TID1S overexpression also decreased ATP levels, a marker of cell viability (Figure 6C, 26% decrease, *n* = 4, *p* < 0.05) and increased mitochondrial fragmentation in fibroblasts (Figure 6D). While lentivirus-mediated TID1L overexpression had no effect on mature frataxin (Supplementary Figure S3A), it resulted in mitochondria fragmentation (Supplementary Figure S3B). These results indicate that TID1L and TID1S have distinct roles in regulating frataxin in fibroblasts.

### 3.5 A peptide targeting TID1S rescues frataxin deficiency and mitochondrial phenotype in FRDA patient-derived skin fibroblasts

**FIGURE 7:**
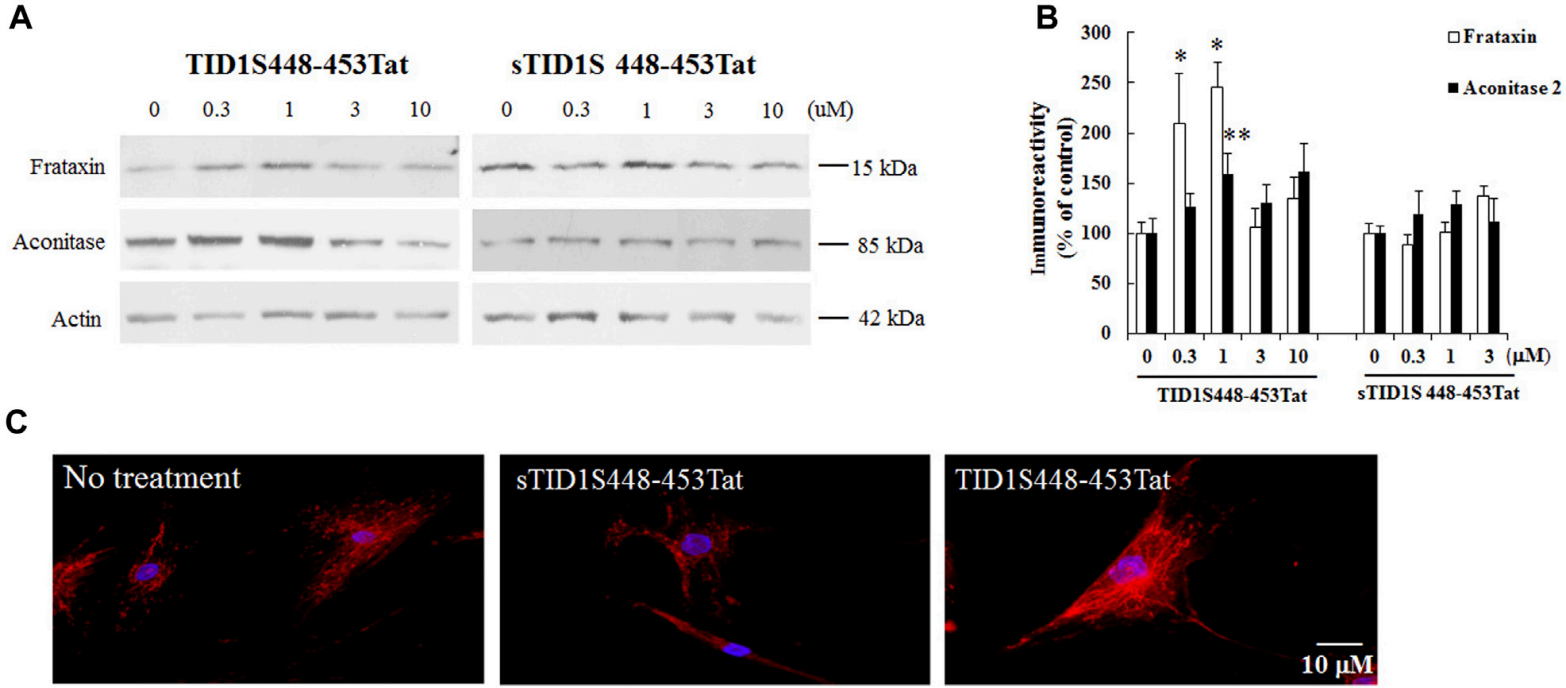
TID1S448-453Tat rescues frataxin deficiency and mitochondrial defect in FRDA patient-derived skin fibroblasts. FRDA patient skin fibroblasts were treated with TID1S448-453Tat and sTID1448-453Tat for 48 h followed by Western blotting analysis or immunofluorescence. Compared with sTID1448-453Tat, TID1S448-453Tat does-dependently increased frataxin levels **(A,B)** (*n* = 5). 1μM TID1S448-453Tat treatment also increased aconitase levels **(A,B)** (*n* = 5) and rescued mitochondrial fragmentation in FRDA patient skin fibroblasts **(C)**. Mitotracker was used to identify mitochondria. ∗*p* < 0.05, ∗∗*p* < 0.01. Data were shown as mean ± SE.

TID1S and TID1L only differ in the C-terminus. TID1L has 33 amino acids unique to its C-terminus while TID1S contains 6 amino acids (Lu et al., 2006). To investigate whether the effect of TID1S on frataxin is mediated by the last 6 amino acids of its C-terminus (448-453), we generated a competing peptide based on the amino acid sequence of TID1S448-453. To make the TID1S448-453 peptide permeable to cell membranes, it was coupled with a peptide from the transactivator of transcription (TAT) of human immunodeficiency virus (Liu et al., 2008). As a negative control, scrambled TID1S448-453 (sTID1S448-453) was also produced. FRDA patient-derived skin fibroblasts were treated with both peptides for 48 h followed by Western blot analysis. In comparison with sTID1S448-453Tat, TID1S448-453Tat dose dependently increased frataxin levels with maximal effect achieved at 1 μM (Figures 7A, B) (146% increase, *n* = 5, *p* < 0.05). 1μM TID1S448-453Tat also significantly increased aconitase levels (Figures 7A, B) (59% increase, *n* = 5, *p* < 0.01) and rescued mitochondrial fractionation (Figure 7C) in FRDA patient fibroblasts.

## 4 Discussion

In the present study, we demonstrate that TID1 is a novel binding partner and regulator of frataxin. In both HEK293 cells and primary cultured human skin fibroblasts, overexpression of TID1S lowers the amounts of mature frataxin. The effect of TID1S on frataxin is mediated by the last 6 amino acids of TID1S as a competing peptide made from these amino acids rescues frataxin deficiency and mitochondrial abnormalities in the FRDA cellular model. Our findings thus identify TID1S as a negative posttranslational regulator of frataxin and a new therapeutic target for FRDA.

TID1L and TID1S differ only at their extreme carboxyl termini but have distinct role in regulating frataxin. While both TID1L and TID1S increase frataxin precursor and decrease mature frataxin in HEK293 cells, only TID1S overexpression decreases mature frataxin in primary cultured fibroblasts. The lack of effect of TID1L in primary cultured fibroblasts could be ascribed to both the altered protein interactome and its limited binding affinity for frataxin (Figures 1C, D). Aside from this, TID1L has a longer residency time in the cytosol due to its interaction with the cytosolic chaperone Hsc70 and its cytosolic substrates such as STAT1 and STAT3, (Lu et al., 2006), whereas TID1S predominates in the mitochondria where it is more likely to interact with and control frataxin. Since TID1S overexpression has no effect on the mitochondrial localization of frataxin precursor and a proteasomal inhibitor, MG132, does not restore intermediate and mature frataxin in HEK293 cells, the TID1S overexpression-generated decrease in mature frataxin is neither due to compromised mitochondrial import nor to proteasomal degradation. Given that frataxin is susceptible to degradation by mitochondrial proteases such as PITRM1 in addition to the proteasome (Rufuni et al., 2011; Nabhan et al., 2015; Hackett et al., 2022), one explanation for this decrease is that TID1S directs frataxin precursor to mitochondrial proteases for destruction. Additionally, unlike GRP75, which facilitates the maturation of frataxin by MPP, TID1S does not interact with MPP. The binding of TID1S to frataxin alone may not only decrease the efficiency of MPP-mediated frataxin maturation but also make the frataxin precursor more accessible to other methods of destruction. It will be interesting to know whether mitochondrial proteases are involved in this process and whether TID1S and GRP75 compete with one another for binding to frataxin, decreasing the effectiveness of GRP75-facilitated frataxin maturation.

Altered TID1 protein levels are noted in some neurodegenerative diseases including Alzheimer’s disease (AD) and Parkinson’s disease (PD). Elevated TID1 protein levels are observed in the hippocampus of both AD patients and Tg2576 mice. This elevation contributes to Aβ42 induced neurotoxicity through reactive oxygen species generation, apoptosis and Aβ production, all of which can be reversed by TID1 knockdown (Zhou et al., 2020). In the 6-OHDA rat model of Parkinson’s disease, a 26 kDa breakdown product of TID1 is widely increased following a 6-OHDA lesion. TID1 protein is also reduced in CAD (CNS-derived catecholaminergic neuronal cell line) cells by the same treatment (Proft et al., 2011). No alteration in heat shock proteins such as Hsp70 is seen in either the *in vitro* or *in vivo* model of Parkinson’s disease (Proft et al., 2011), indicating a critical role of TID1 in the pathogenesis of neurodegenerative diseases. Our results demonstrate that both TID1L and TID1S are increased in the affected tissues of frataxin knockdown mice upon subacute frataxin deficiency. TID1L is widely elevated whereas TID1S is only increased in specific tissues, including the heart and skeletal muscle, suggesting tissue specific effects of frataxin deficiency on TID1 splice variants levels. TID1S elevation could result in a further decrease in frataxin levels leading to exacerbation of pathological changes in FRDA, forming a vicious cycle. While TID1L overexpression has no effect on mature frataxin in primary cultured fibroblasts, it causes mitochondrial fragmentation, just like TID1S. TID1L-caused mitochondrial fragmentation is Dynamin-related protein 1 (Drp1) dependent and associated with reduced cell viability (Elwi et al., 2012). Consistent with increased TID1L, in the cerebellum of frataxin knockdown mice increased activation of Drp1 and structural changes in mitochondria were observed in the cerebellum of one FRDA mouse model (Mercado-Ayón et al., 2022). Similar results are also observed *in vitro* in frataxin deficient fibroblasts (Johnson et al., 2021), suggesting that TID1L elevation could also contribute to pathological changes in FRDA. Although the mechanism by which frataxin deficiency increases TID1 is yet unclear, one possibility is that frataxin deficiency-caused oxidative stress activates the integrated stress response (Vásquez-Trincado et al., 2022), consequently affecting TID1 levels.

In contrast to TID1L elevation upon acute and subacute frataxin deficiency, TID1L is significantly decreased in FRDA patient-derived cells, suggesting that chronic frataxin deficiency causes the opposite changes in TID1L. Consistent with previous reports in Hela cells (Elwi et al., 2012), TID1L decreases also cause mitochondrial fragmentation in fibroblasts (Supplementary Figure S4), suggesting that both TID1L elevation and decrease can contribute to pathological changes in FRDA. An optimal concentration of TID1 may be needed for its physiological effects. The decrease in TID1L levels may reflect decreased mitochondrial numbers (Huang et al., 2013; Vásquez-Trincado et al., 2022) as multiple proteins including GRP75 are lowered in FRDA patient cells (Dong et al., 2019). The easy accessibility of such patient cells suggests that TID1L decrease could serve as a biomarker for FRDA.

Our result that TID1S448-453 rescuing frataxin deficiency and mitochondrial defects in FRDA fibroblasts offers a potential small molecule therapeutic candidate for FRDA. Although more research is needed to determine whether TID1S448-453 can effectively restore the frataxin levels and phenotype of FRDA neurons and cardiomyocytes, the most severely affected tissues, as well as animal models, its small molecular weight and ease of modification provide a new avenue for therapeutic development. In addition, TID1S448-453 increases aconitase 2 protein levels, suggesting a potential impact on mitochondrial biogenesis. TID1S448-453 may be beneficial for a number of neurodegenerative diseases, including FRDA, which have a mitochondrial biogenesis deficit (Calkins et al., 2011; Uittenbogaard and Chiaramello, 2014; Golpich et al., 2017) and aconitase 2 deficient disorders (Spiegel et al., 2012; Bouwkamp et al., 2018; Neumann et al., 2020; Park et al., 2020).

## Data Availability

The original contributions presented in the study are included in the article/Supplementary Material, further inquiries can be directed to the corresponding authors.

## References

[B1] Al-Mahdawi S. Pinto R. M. Varshney D. Lawrence L. Lowrie M. B. Hughes S. (2006). GAA repeat expansion mutation mouse models of Friedreich ataxia exhibit oxidative stress leading to progressive neuronal and cardiac pathology. Genomics 88, 580–590. 10.1016/j.ygeno.2006.06.015 16919418 PMC2842930

[B45] Bae M. K. Jeong J. W. Kim S. H. Kim S. Y. Kang H. J. Kim D. M. (2005). Tid-1 interacts with the von Hippel-Lindau protein and modulates angiogenesis by destabilization of HIF-1alpha. Cancer Res. 65 (7), 2520–2525. 10.1158/0008-5472.CAN-03-2735 15805242

[B2] Bouwkamp C. G. Afawi Z. Fattal-Valevski A. Krabbendam I. E. Rivetti S. Masalha R. (2018). ACO2 homozygous missense mutation associated with complicated hereditary spastic paraplegia. Neurol. Genet. 4, e223. 10.1212/NXG.0000000000000223 29577077 PMC5863690

[B3] Calkins M. J. Manczak M. Mao P. Shirendeb U. Reddy P. H. (2011). Impaired mitochondrial biogenesis, defective axonal transport of mitochondria, abnormal mitochondrial dynamics and synaptic degeneration in a mouse model of Alzheimer's disease. Hum. Mol. Genet. 20, 4515–4529. 10.1093/hmg/ddr381 21873260 PMC3209824

[B4] Campuzano V. Montermini L. Moltò M. D. Pianese L. Cossée M. Cavalcanti F. (1996). Friedreich’s ataxia: autosomal recessive disease caused by an intronic GAA triplet repeat expansion. Science 271, 1423–1427. 10.1126/science.271.5254.1423 8596916

[B5] Chen C. Y. Chiou S. H. Huang C. Y. Jan C. I. Lin S. C. Hu W. Y. (2009). Tid1 functions as a tumour suppressor in head and neck squamous cell carcinoma. J. Pathol. 219 (3), 347–355. 10.1002/path.2604 19681071

[B6] Cheng H. Cenciarelli C. Shao Z. Vidal M. Parks W. P. Pagano M. (2001). Human T cell leukemia virus type 1 Tax associates with a molecular chaperone complex containing hTid-1 and Hsp70. Curr. Biol. 11 (22), 1771–1775. 10.1016/s0960-9822(01)00540-1 11719219

[B7] Clark E. Butler J. S. Isaacs C. J. Napierala M. Lynch D. R. (2017). Selected missense mutations impair frataxin processing in Friedreich ataxia. Ann. Clin. Transl. Neurol. 4, 575–584. 10.1002/acn3.433 28812047 PMC5553228

[B44] Deutsch E. C. Santani A. B. Perlman S. L. Farmer J. M. Stolle C. A. Marusich M. F. (2010). A rapid, noninvasive immunoassay for frataxin: utility in assessment of Friedreich ataxia. Mol. Genet. Metab. 101 (2–3), 238–245. 10.1016/j.ymgme.2010.07.001 20675166 PMC2996613

[B8] Dong Y. N. McMillan E. Clark E. M. Lin H. Lynch D. R. (2019). GRP75 overexpression rescues frataxin deficiency and mitochondrial phenotypes in Friedreich ataxia cellular models. Hum. Mol. Genet. 28, 1594–1607. 10.1093/hmg/ddy448 30590615 PMC6494971

[B9] Dong Y. N. Mesaros C. Xu P. Mercado-Ayón E. Halawani S. Ngaba L. V. (2022). Frataxin controls ketone body metabolism through regulation of OXCT1. PNAS Nexus 1, pgac142. 10.1093/pnasnexus/pgac142 36016708 PMC9396447

[B10] Elwi A. N. Lee B. Meijndert H. C. Braun J. E. A. Kim S. W. (2012). Mitochondrial chaperone DnaJA3 induces Drp1-dependent mitochondrial fragmentation. Int. J. Biochem. Cell Biol. 44, 1366–1376. 10.1016/j.biocel.2012.05.004 22595283

[B11] Golpich M. Amini E. Mohamed Z. Ali R. A. Ibrahim N. M. Ahmadiani A. (2017). Mitochondrial dysfunction and biogenesis in neurodegenerative diseases: pathogenesis and treatment. CNS Neurosci. Ther. 23, 5–22. 10.1111/cns.12655 27873462 PMC6492703

[B12] Hackett P. T. Jia X. Li L. Ward D. M. (2022). Posttranslational regulation of mitochondrial frataxin and identification of compounds that increase frataxin levels in Friedreich's ataxia. J. Biol. Chem. 298, 101982. 10.1016/j.jbc.2022.101982 35472330 PMC9127368

[B13] Hayashi M. Imanaka-Yoshida K. Yoshida T. Wood M. Fearns C. Tatake R. J. (2006). A crucial role of mitochondrial Hsp40 in preventing dilated cardiomyopathy. Nat. Med. 12, 128–132. 10.1038/nm1327 16327803

[B14] Hendrick J. P. Langer T. Davis T. A. Wiedmann M. (1993). Control of folding and membrane translocation by binding of the chaperone DnaJ to nascent polypeptides. Proc. Natl. Acad. Sci. U. S. A. 90, 10216–10220. 10.1073/pnas.90.21.10216 8234279 PMC47745

[B15] Huang M. L. H. Sivagurunathan S. Ting S. Jansson P. J. Austin C. J. D. Kelly M. (2013). Molecular and functional alterations in a mouse cardiac model of Friedreich ataxia activation of the integrated stress response, eIF2a phosphorylation, and the induction of downstream targets. Am. J. Pathol. 183, 745–757. 10.1016/j.ajpath.2013.05.032 23886890

[B43] Johnson J. Mercado-Ayón E. Clark E. Lynch D. Lin H. (2021). Drp1-dependent peptide reverse mitochondrial fragmentation, a homeostatic response in Friedreich ataxia. Pharmacol. Res. Perspect. 9 (3), e00755. 10.1002/prp2.755 33951329 PMC8099044

[B16] Linnoila J. Wang Y. Yao Y. Wang Z. Z. (2008). A mammalian homolog of Drosophila tumorous imaginal discs, Tid1, mediates agrin signaling at the neuromuscular junction. Neuron 60, 625–641. 10.1016/j.neuron.2008.09.025 19038220 PMC3225410

[B17] Liu T. Daniels C. K. Cao S. (2012) Comprehensive review on the HSC70 functions, interactions with related molecules and involvement in clinical diseases and therapeutic potential. Pharmacol. Ther. 136, 354–374. 10.1016/j.pharmthera.2012.08.014 22960394

[B18] Liu X. J. Gingrich J. R. Vargas-Caballero M. Dong Y. N. Sengar A. Beggs S. (2008). Treatment of inflammatory and neuropathic pain by uncoupling Src from the NMDA receptor complex. Nat. Med. 14, 1325–1332. 10.1038/nm.1883 19011637 PMC3616027

[B19] Lo J. F. Hayashi M. Woo-Kim S. Tian B. Huang J. F. Fearns C. (2004). Tid1, a cochaperone of the heat shock 70 protein and the mammalian counterpart of the Drosophila tumor suppressor l(2)tid, is critical for early embryonic development and cell survival. Mol. Cell Biol. 24 (6), 2226–2236. 10.1128/MCB.24.6.2226-2236.2004 14993262 PMC355836

[B20] Lodi R. Cooper J. M. Bradley J. L. Manners D. Styles P. Taylor D. J. (1999). Deficit of *in vivo* mitochondrial ATP production in patients with Friedreich ataxia. Proc. Natl. Acad. Sci. U. S. A. 96, 11492–11495. 10.1073/pnas.96.20.11492 10500204 PMC18061

[B21] Lu B. Garrido N. Spelbrink J. N. Suzuki C. K. (2006). Tid1 isoforms are mitochondrial DnaJ-like chaperones with unique carboxyl termini that determine cytosolic fate. J. Biol. Chem. 281, 13150–13158. 10.1074/jbc.M509179200 16531398

[B22] Mercado-Ayón E. Warren N. Halawani S. Rodden L. N. Ngaba L. Dong Y. N. (2022). Cerebellar pathology in an inducible mouse model of Friedreich ataxia. Front. Neurosci. 16, 819569. 10.3389/fnins.2022.819569 35401081 PMC8987918

[B23] Nabhan J. F. Gooch R. L. Piatnitski E. L. Pierce B. Bulawa C. E. (2015). Perturbation of cellular proteostasis networks identifies pathways that modulate precursor and intermediate but not mature levels of frataxin. Sci. Rep. 5, 18251. 10.1038/srep18251 26671574 PMC4680912

[B24] Neumann M. A. Grossmann D. Schimpf-Linzenbold S. Dayan D. Stingl K. Ben-Menachem R. (2020). Haploinsufficiency due to a novel ACO2 deletion causes mitochondrial dysfunction in fibroblasts from a patient with dominant optic nerve atrophy. Sci. Rep. 10, 16736. 10.1038/s41598-020-73557-4 33028849 PMC7541502

[B25] Ng A. C. Baird S. D. Screaton R. A. (2014). Essential role of TID1 in maintaining mitochondrial membrane potential homogeneity and mitochondrial DNA integrity. Mol. Cell Biol. 34 (8), 1427–1437. 10.1128/MCB.01021-13 24492964 PMC3993590

[B26] Park J. S. Kim M. J. Kim S. Y. Lim B. C. Seong M. W. (2020). Novel compound heterozygous ACO2 mutations in an infant with progressive encephalopathy: a newly identified neurometabolic syndrome. Brain Dev. 42, 680–685. 10.1016/j.braindev.2020.07.003 32713659

[B27] Proft J. Faraji J. Robbins J. C. Zucchi F. C. R. Zhao X. Metz G. A. (2011). Identification of bilateral changes in TID1 expression in the 6-OHDA rat model of Parkinson's disease. PLoS One 6 (10), e26045. 10.1371/journal.pone.0026045 22016808 PMC3189242

[B28] Puccio H. Simon D. Cossée M. Criqui-Filipe P. Tiziano F. Melki J. (2001). Mouse models for Friedreich ataxia exhibit cardiomyopathy, sensory nerve defect and Fe–S enzyme deficiency followed by intramitochondrial iron deposits. Nat. Genet. 27, 181–186. 10.1038/84818 11175786

[B29] Rötig A. Lonlay P. Chretien D. Foury F. Koenig M. Sidi D. (1997). Aconitase and mitochondrial iron–sulphur protein deficiency in Friedreich ataxia. Nat. Genet. 17, 215–217. 10.1038/ng1097-215 9326946

[B30] Rufini A. Fortuni S. Arcuri G. Condò I. Serio D. Incani O. (2011). Preventing the ubiquitin-proteasome-dependent degradation of frataxin, the protein defective in Friedreich’s ataxia. Hum. Mol. Genet. 20, 1253–1261. 10.1093/hmg/ddq566 21216878

[B31] Silver P. A. Way J. C. (1993). Eukaryotic DnaJ homologs and the specificity of Hsp70 activity. Cell 74, 5–6. 10.1016/0092-8674(93)90287-z 8334705

[B32] Spiegel R. Pines O. Ta-Shma A. Burak E. Shaag A. Halvardson J. (2012). Infantile cerebellar-retinal degeneration associated with a mutation in mitochondrial aconitase, ACO2. Am. J. Hum. Genet. 90, 518–523. 10.1016/j.ajhg.2012.01.009 22405087 PMC3309186

[B33] Stehling O. Elsässer H. P. Brückel B. Mühlenhoff U. Lill R. (2004). Iron–sulfur protein maturation in human cells: evidence for a function of frataxin. Hum. Mol. Genet. 13, 3007–3015. 10.1093/hmg/ddh324 15509595

[B34] Strawser C. Schadt K. Hauser L. McCormick A. Wells M. Larkindale J. (2017). Pharmacological therapeutics in Friedreich ataxia: the present state. Expert Rev. Neurother. 17 (9), 895–907. 10.1080/14737175.2017.1356721 28724340

[B35] Syken J. De-Medina T. Münger K. (1999). TID1, a human homolog of the Drosophila tumor suppressor l(2)tid, encodes two mitochondrial modulators of apoptosis with opposing functions. Proc. Natl. Acad. Sci. U. S. A. 96 (15), 8499–8504. 10.1073/pnas.96.15.8499 10411904 PMC17545

[B36] Syken J. Macian F. Agarwal S. Rao A. Münger K. (2003). TID1, a mammalian homologue of the drosophila tumor suppressor lethal(2) tumorous imaginal discs, regulates activation-induced cell death in Th2 cells. Oncogene 22, 4636–4641. 10.1038/sj.onc.1206569 12879007

[B37] Tarunina M. Alger L. Chu G. Munger K. Gudkov A. Jat P. S. (2004). Functional genetic screen for genes involved in senescence: role of Tid1, a homologue of the Drosophila tumor suppressor l(2)tid, in senescence and cell survival. Mol. Cell Biol. 24, 10792–10801. 10.1128/MCB.24.24.10792-10801.2004 15572682 PMC533960

[B38] Trentin G. A. Yin X. Tahir S. Lhotak S. Farhang-Fallah J. Li Y. (2001). A mouse homologue of the Drosophila tumor suppressor l(2)tid gene defines a novel Ras GTPase-activating protein (RasGAP)-binding protein. J. Biol. Chem. 276, 13087–13095. 10.1074/jbc.M009267200 11116152

[B39] Uittenbogaard M. Chiaramello A. (2014). Mitochondrial biogenesis: a therapeutic target for neurodevelopmental disorders and neurodegenerative diseases. Curr. Pharm. Des. 20, 5574–5593. 10.2174/1381612820666140305224906 24606804 PMC4823001

[B40] Vásquez-Trincado C. Dunn J. Han J. I. Hymms B. Tamaroff J. Patel M. (2022). Frataxin deficiency lowers lean mass and triggers the integrated stress response in skeletal muscle. JCI Insight 7, e155201. 10.1172/jci.insight.155201 35531957 PMC9090249

[B41] Zarse K. Schulz T. J. Birringer M. Ristow M. (2007). Impaired respiration is positively correlated with decreased life span in *Caenorhabditis elegans* models of Friedreich ataxia. FASEB J. 21, 1271–1275. 10.1096/fj.06-6994com 17215485

[B42] Zhou C. Taslima F. Abdelhamid M. Kim S. W. Akatsu H. Michikawa M. (2020). Beta-amyloid increases the expression levels of Tid1 responsible for neuronal cell death and amyloid beta production. Mol. Neurobiol. 57, 1099–1114. 10.1007/s12035-019-01807-2 31686372

