# Supplementary material for: A peptide derived from TID1S rescues frataxin deficiency and mitochondrial defects in FRDA cellular models
Source: Front Pharmacol. 2024 Mar 1;15:1352311. doi: 10.3389/fphar.2024.1352311 (PMC10940384; doi:10.3389/fphar.2024.1352311)
Supplement: Supplementary file 1 [file DataSheet1.docx]

Supplemental material for

**A peptide derived from TID1S rescues frataxin deficiency and mitochondrial defects in FRDA cellular models**

Yi Na Dong^1 ,2*^, Lucie Vanessa Ngaba^1^, Jacob An^1^, Miniat W Adeshina^1^, Nathan Warren^1^, Johnathan Wong^1^, David R. Lynch^1, 2*^

Yi Na Dong: [Dongy@chop.edu](mailto:Dongy@chop.edu)

David R. Lynch: lynchd@pennmedicine.upenn.edu

Table S1. TID1 (DNAJA3) is associated with frataxin.

**Supplemental Figure 1**. Effect of frataxin knockdown on TID1L protein levels. Representative blots and bar graph show decreased frataxin and increased TID1L protein levels in human skin fibroblasts transfected with frataxin siRNA for 5 days (A and B) (n=6) as well as in the homogenates of cerebellum (C and D) (n=6) and heart (E and F) (n=6) from frataxin knockdown mice induced with doxycycline for 4 weeks. **P*<0.05, ***P*<0.01. Data were shown as mean±SE.

**Supplemental Figure 2**. TID1S is not highly expressed in buccal cells, platelets and PBMCs. Buccal cells, platelets and PBMCs collected from healthy individuals were lysed and subject to Western blot analysis. Compared with TID1L, the TID1S expression is minimal.

**Supplemental Figure 3**. Effect of TIDL overexpression on mature frataxin and mitochondrial morphology. Human skin fibroblasts were transduced with lentivirus carrying pHAGE-TID1L gene or vector control for 5 days followed by Western blot analysis or immunofluorescence. TID1L transduction had no effect on mature frataxin (A) (n=4) but led to mitochondrial fragmentation (B). Immunofluorescence was performed using an anti-TID1L antibody and mitochondria was identified with mitotracker.

**Supplemental Figure 4**. Effects of TID1L knockdown on mitochondrial morphology. Human skin fibroblasts transfected with control or TID1L siRNA were subjected to Western blotting or immunofluorescence. Fibroblasts transfected with TID1L siRNA displayed decreased TID1L levels (A and B) (n=4) and fragmented mitochondria (C) in comparison with control siRNA ***P*<0.01. Data were shown as mean±SE. Scale bar=50 μM.
